# Quantitative trait loci for yield and grain plumpness relative to maturity in three populations of barley (*Hordeum vulgare* L.) grown in a low rain-fall environment

**DOI:** 10.1371/journal.pone.0178111

**Published:** 2017-05-23

**Authors:** Bulti Tesso Obsa, Jason Eglinton, Stewart Coventry, Timothy March, Maxime Guillaume, Thanh Phuoc Le, Matthew Hayden, Peter Langridge, Delphine Fleury

**Affiliations:** 1 Australian Centre for Plant Functional Genomics, School of Agriculture, Food and Wine, Waite Research Institute, The University of Adelaide, Glen Osmond, South Australia, Australia; 2 School of Agriculture, Food and Wine, Waite Research Institute, The University of Adelaide, Glen Osmond, South Australia, Australia; 3 Montpellier SupAgro, Montpellier, France; 4 Department of Plant Protection, College of Agriculture & Applied Biology, Can Tho University, Can Tho, Vietnam; 5 Department of Economic Development, Jobs, Transport and Resources, Agribio, La Trobe University, Bundoora, Victoria, Australia; New South Wales Department of Primary Industries, AUSTRALIA

## Abstract

Identifying yield and grain plumpness QTL that are independent of developmental variation or phenology is of paramount importance for developing widely adapted and stable varieties through the application of marker assisted selection. The current study was designed to dissect the genetic basis of yield performance and grain plumpness in southern Australia using three doubled haploid (DH) populations developed from crosses between adapted parents that are similar in maturity and overall plant development. Three interconnected genetic populations, Commander x Fleet (CF), Commander x WI4304 (CW), and Fleet x WI4304 (FW) developed from crossing of Australian elite barley genotypes, were used to map QTL controlling yield and grain plumpness. QTL for grain plumpness and yield were analysed using genetic linkage maps made of genotyping-by-sequencing markers and major phenology genes, and field trials at three drought prone environments for two growing seasons. Seventeen QTL were detected for grain plumpness. Eighteen yield QTL explaining from 1.2% to 25.0% of the phenotypic variation were found across populations and environments. Significant QTL x environment interaction was observed for all grain plumpness and yield QTL, except *QPlum*.*FW-4H*.*1* and *QYld*.*FW-2H*.*1*. Unlike previous yield QTL studies in barley, none of the major developmental genes, including *Ppd-H1*, *Vrn-H1*, *Vrn-H2* and *Vrn-H3*, that drive barley adaption significantly affected grain plumpness and yield here. Twenty-two QTL controlled yield or grain plumpness independently of known maturity QTL or genes. Adjustment for maturity effects through co-variance analysis had no major effect on these yield QTL indicating that they control yield *per se*.

## Introduction

The average yield of Australian barley is 2 t/ha [1], which is below the world average of 3 t/ha [2]. Barley production in southern Australia is particularly constrained by cyclic and terminal drought in addition to a number of biotic, abiotic and physiochemical subsoil stresses. Yield is a complex quantitative trait whose expression is highly influenced by the environment and agronomic management. This makes phenotype-based selection slow and unreliable, especially under environments where multiple abiotic stresses prevail. Developing barley varieties with improved and stable yield in such environments is expected to be more challenging with ongoing climate change, thus requiring substantial changes in agronomic practices and crop improvement approaches [3].

In addition to high yield, barley varieties need to meet minimum grain plumpness standards to be marketed to different end users. Grain plumpness is the minimum retention (% by weight) of grain above a 2.5 mm slotted screen, the specifications for the MALT1, MALT2 and MALT3 grades being 70%, 62% and 58% respectively in Australia [4]. Increased grain plumpness is associated with important quality attributes for malting barley such as higher malt extract and moderate grain protein [5]. Grain plumpness is affected by the genotype and the environment [6] and is highly heritable with values of 88% to 96% reported under variable environments [7], indicating the potential for improvement. Grain plumpness is determined by pre-anthesis plant development related traits that affect assimilate accumulation and post-anthesis physiological traits affecting assimilate supply to the developing grain [6]. Farmers aim to maximise yield and grain plumpness agronomically by optimising pre-anthesis biomass production and flowering time for their environment. Genetics can be used to achieve this through improved water use efficiency, biomass production and partitioning to the grain, and by selecting for abiotic and biotic stress tolerance.

Quantitative trait loci (QTL) mapping is an important step towards development of reliable markers for marker assisted selection. QTL mapping studies for yield and other agronomic traits have been conducted under different environments using different genetic backgrounds in barley [8–23]. A usual problem in yield QTL studies under dry climate is the absence of consideration for phenology or maturity as a confounding factor. Plants can escape drought stress by completing its cycle before water deficit becomes severe [24]. A short cycle is particularly advantageous in environments with terminal drought stress as in Australian climate. As a result, plant maturity strongly influences grain yield under dry conditions. Frequently the reported yield-related QTL were associated with the major phenology genes such as the vernalization requirement genes (*Vrn-H1*, *Vrn-H2*, and *Vrn-H3*) [25], the photoperiod response genes (*Ppd-H1* and *Ppd-H2*) and the earliness *per se* (*EPS2*) locus [20, 26]. QTL have been mapped for different aspects of grain size including grain weight, grain length, grain width, and grain width to length ratio [11, 21]. Genomic regions affecting barley grain weight and size in different international and Australian mapping populations have been summarized in [6]. Most of these QTL were associated with loci influencing plant development, mainly with *Ppd-H1*, the *Eps2*, and the semi-dwarfing gene *Denso* (*sdw1*).

*Ppd-H1* and *Vrn-H1* are the two major genes affecting flowering time in barley and have significant effects on agronomic traits including yield components [27]. An important gene family called *FLOWERING LOCUS T* (*FT*) induces or represses flowering in plants; this includes the barley genes *HvFT1/Vrn-H3* [28], *HvFT3/Ppd-H2*, *TERMINAL FLOWER 1* (*HvTFL1*) [29] and *CENTRORADIALIS* (*HvCEN*) gene, which is the candidate gene for *EPS2* [30]. *EPS2* affects flowering time and other agronomic traits including tiller biomass, tiller grain weight, ear grain number, and plant height [31]. Other phenology genes are associated with circadian rhythm such as the barley *CONSTANS* gene (*HvCO1* and *HvCO2*) [32], the *GIGANTEA* (*HvGI*) [33] and the red/far-red light *PHYTOCHROMES* with the barley genes, *HvPhyA*, *HvPhyB* and *HvPhyC* [34], and the *APETALA2* (*HvAP2*) gene that control inflorescence development [35].

Breeding for a shortened crop cycle has been a very successful strategy in Mediterranean climate. In Australia, barley maturity has already been optimised by breeders so that the plant’s phenology matches its environment. This study therefore aimed to identify novel QTL for yield and grain plumpness that were not due to phenology. Identifying such QTL that are independent of developmental variation or phenology is important for developing widely adapted and stable varieties through the application of marker assisted selection. The current study was designed to dissect the genetic basis of yield performance and grain plumpness in southern Australia using three doubled haploid (DH) populations developed from crosses between adapted parents that are similar in maturity and overall plant development.

## Materials and methods

### Plant material and phenotyping

Three doubled haploid (DH) populations of barley developed from reciprocal crosses among three Australian lines were used [36]. The parental lines include two elite varieties (Commander and Fleet) and one advanced breeding line (WI4304). Commander (Keel/Sloop//Galaxy) is a malting variety with large grain size and is high yielding in southern Australia. WI4304 (Riviera/ (Puffin/Chebec)-50//Flagship) is a malting quality breeding line with high osmotic adjustment and high net photosynthesis under drought conditions [37]. Fleet (Mundah/Keel//Barque) is a feed variety characterized by high water use efficiency, long coleoptile, and adaptation to deep sandy soils. The parents have similar maturity which enable us to dissect the genetic basis of yield in the Australian environment with minimal confounding effect of phenology. The populations include 229 lines from the Commander x Fleet (CF), 228 lines from the Commander x WI4304 (CW), and 299 lines from the Fleet x WI4304 (FW).

Data were collected from six field trials in South Australia at Minnipa Research Centre (MRC), Roseworthy Campus of the University of Adelaide (RAC), and Swan Hill (SWA) in 2012 and 2013 cropping seasons. Each field trial was an un-replicated design with gridded checks of the parents and two reference varieties (Hindmarsh and Capstan) every eight plots. Plot size were 4 m^2^ in Roseworthy, 5 m^2^ in Minnipa and 7.5 m^2^ in Swan Hill. The grain was machine harvested at physiological maturity from standard breeder’s plots and yields were converted into tonnes per hectare. Grain plumpness was obtained from the plot yields using a seed cleaning machine with a 2.5 mm slotted screen, and expressed as retention (% by weight) based on the specifications given by Grain Trade Australia (GTA 2014). Maturity was assessed as decimal growth stage based on Zadoks scale [38] which is a standardized scale of cereal development divided into ten principal growth stages from germination to ripening. Zadoks stage was recorded per plot, once per trial, when most plants were between booting and heading stages (Z41-Z59). The data for the six trials on these three barley populations were fully described in [36].

### Statistical analysis of phenotypic data

A multi-stage analysis was performed using the regular grid spatial design and Residual Maximum Likelihood (REML) variance components model in GenStat Version 17 (VSN International Ltd, UK). Spatial models (random and linear row and column effects) were fitted for each experiment using plotted variograms to identify spatial co-variance structures [39]. Best Linear Unbiased Predictors (BLUPs) were generated using genotype random effects to estimate the generalized heritability [40]. Anova and least significant difference (LSD) were used for comparing parental lines (Table 1).

**Table 1:** Summary statistics based on BLUPs for yield and grain plumpness for three populations and variation in the parents at six different environments.

	Yield (t/ha)						Grain plumpness (% >2.5 mm)				
	MRC12	MRC13	RAC12	RAC13	SWA12	SWA13	MRC13	RAC12	RAC13	SWH12	SWH13
Commander	1.31^a^	3.83^a^	3.45	3.11^b^	2.81^a^	2.61^a^	82.3^b^	80.6	85.4^c^	83.9^b^	92.6^b^
Fleet	1.34^a^	3.92^a^	3.32	3.33^b^	2.82^a^	2.59^a^	86.4^a^	82.3	93.6^a^	90.1^a^	95.9^a^
WI4304	1.00^b^	3.54^b^	3.41	3.73^a^	2.34^b^	2.15^b^	78.7^c^	81	91.2^b^	77.2^c^	91.5^b^
F-probability	**<0.001**	**<0.001**	**0.54**^**ns**^	**<0.001**	**<0.001**	**<0.001**	**<0.001**	**0.80**^**ns**^	**<0.001**	**<0.001**	**<0.001**
CF mean	1.26	3.44	3.20	2.91	2.69	2.65	82.8	83.4	93.7	77.6	89.6
CF minimum	0.70	2.62	1.80	1.55	1.82	0.56	57.8	56.3	82.5	40.4	67.4
CF maximum	1.62	4.12	4.06	4.17	3.25	3.72	96.4	96.5	98.5	97.5	97.8
s.d.	0.15	0.26	0.40	0.46	0.24	0.38	7.2	7.7	3.1	11.5	5.3
Heritability	**0.53**	**0.51**	**0.68**	**0.59**	**0.7**	**0.39**	**0.71**	**0.78**	**0.66**	**0.85**	**0.71**
CW mean	1.10	3.37	3.53	3.06	2.49	2.30	78.9	73.8	91.3	79	86.7
CW minimum	0.56	2.56	2.54	1.35	1.58	1.46	48.2	38.3	76.7	38.9	53.2
CW maximum	1.58	4.04	4.40	4.41	3.52	3.50	96.2	94.3	97.9	98.7	98.2
s.d.	0.19	0.28	0.36	0.51	0.32	0.36	9.0	11.1	3.4	11.6	7.2
Heritability	**0.71**	**0.76**	**0.86**	**0.59**	**0.82**	**0.65**	**0.83**	**0.82**	**0.76**	**0.85**	**0.78**
FW mean	1.07	3.44	3.13	3.08	2.45	2.07	83.7	84.9	89.4	74.7	90.9
FW minimum	0.25	1.25	0.50	0.68	0.58	0.37	65.3	53.3	60.6	39.2	71.9
FW maximum	1.56	4.16	4.25	4.10	3.28	2.77	96.7	97.9	98.0	96.9	98.9
s.d.	0.21	0.36	0.40	0.39	0.34	0.29	6.9	7.9	5.82	12.6	4.2
Heritability	**0.74**	**0.75**	**0.77**	**0.65**	**0.57**	**0.67**	**0.64**	**0.87**	**0.65**	**0.79**	**0.81**

s.d. = standard deviation; ^a^, ^b^ and ^c^ are the groups from LSD test; ns = not significant. MRC12 = Minnipa 2012, MRC13 = Minnipa 2013, RAC12 = Roseworthy 2012, RAC13 = Roseworthy 2013, SWH2 = Swan Hill 2012, SWH13 = Swan Hill 2013; CF = Commander x Fleet, CW = Commander x WI4304, FW = Fleet x WI4304.

Best Linear Unbiased Estimates (BLUEs) were generated using genotype fixed effects [41] for QTL and multi-environment analysis since BLUPs are inappropriately scaled by their individual environment heritability and variance estimates [42]. The inverse of the variance matrix of means from each environment was used to generate weights for use in the multi-environment analysis to account for variance heterogeneity. The best model for comparison of across environment covariation was selected based on Schwarz Information Criteria [43]. Genetic correlations among environments for yield were generated from the multi-environment model using the variance-covariance matrix of the selected best model [42]. Pearson’s correlation coefficients (r) were computed to assess the association of yield and grain plumpness with maturity as reported in [36].

### Genotyping and genetic maps

We used the genetic maps described in [36] including 2,178 GBS markers and 7 phenology genes in CF, 2,892 GBS markers and 8 phenology genes in CW, and 2,252 GBS markers and 5 phenology genes in the FW population. The location of GBS markers on barley genome were obtained by BLASTN the tag sequences versus the IBSC barley RefSeq v1.0 of the map-based sequence of cultivar Morex (http://webblast.ipk-gatersleben.de/registration/) [44, 45] and are provided in S1 Table.

The mapped phenology genes were photoperiod response gene (*Ppd-H1*), vernalisation requirement gene (*Vrn-H2*), *APETALA2* (*HvAP2*), *FLOWERING LOCUS T* (*HvFT5*), *TERMINAL FLOWER 1* (*HvTFL1*), *CONSTANS* (*HvCO1*, *HvCO2*), *GIGANTEA* (*HvGI*), the red/far-red light phytochromes genes (*HvPhyB* and *HvPhyC*). The primers used for detecting presence/absence of *Vrn-H2* and for detecting SNP in *HvCO2* and *Ppd-H1* by high-resolution melting curve method were provided in supplementary file of [36]. KASP assays of *HvAP2*, *HvCO1*, *HvFT5*, *HvGI*, *HvPhyB*, *HvPhyC* and *HvTFL1*, were provided by LGC genomics; the sequences are in supplemental file, S1 File. Polymorphism for all phenology genes among the three genetic populations are provided in S2 Table. The markers for *Vrn-H1*, *HvFT1/Vrn-H3*, *HvFT2*, *HvFT3/Ppd-H2* and *HvFT4* from LGC genomics were found monomorphic in the populations (data not shown).

The SNP marker P135A described in [30] for *HvCEN* was found to be monomorphic between the parental lines so we sequenced *HvCEN* in our material. The genomic sequence of *HvCEN* was retrieved from morex_contig_274284 identified by BLASTn analysis of JX648176 sequence from [30] versus the whole genome sequence assembly 3 of cv Morex [44]. Primers were designed to amplify 2,795 bp of *HvCEN* covering of the 5’ upstream region, exons, introns and the 3’ downstream region in Commander, Fleet and WI4304 (S3 and S4 Tables). The PCR fragments were sequenced using the BigDyeTM sequencing chemistry (Applied Biosystems, Perkin Elmer, Weiterstadt, Germany) followed by fluorescent Sanger capillary separation. Sanger sequences were trimmed and merged using the Pairwise Alignment tool of Geneious software (Biomatters Limited, Auckland, New Zealand) that uses the global alignment algorithm [46]. The *HvCEN* sequences were then aligned using Clustalw to identify polymorphism between parental lines. A total of 7 SNP were found between Commander or Fleet and WI4304 (S1 Fig). A KBioscience Competitive Allele-Specific Polymerase chain reaction (KASP) assay was designed using Kraken software to target the intron 3 SNP and named HvCEN_1780. CW and FW populations were genotyped using the KASP primers described in S3 Table and the protocol from LGC genomics (http://www.lgcgroup.com/). HvCEN_1780 marker was added to the linkage maps using MSTmap for R [47] (S2 Fig).

### QTL analysis

QTL analysis of yield and grain plumpness was performed using the generated BLUEs and the updated genetic linkage maps described above. The best variance-covariance model selected in the phenotypic analysis step was used for multi-environment QTL analysis. A genome wide scan to detect candidate QTL positions was performed using Simple Interval Mapping (SIM) [48] followed by Composite Interval Mapping (CIM) [49], in which the QTL detected by SIM were used as cofactors. A genome-wide significance level of α = 0.05 was used as a threshold to reject the null hypothesis of no QTL effect based on the method of [50].

**Fig 1:**
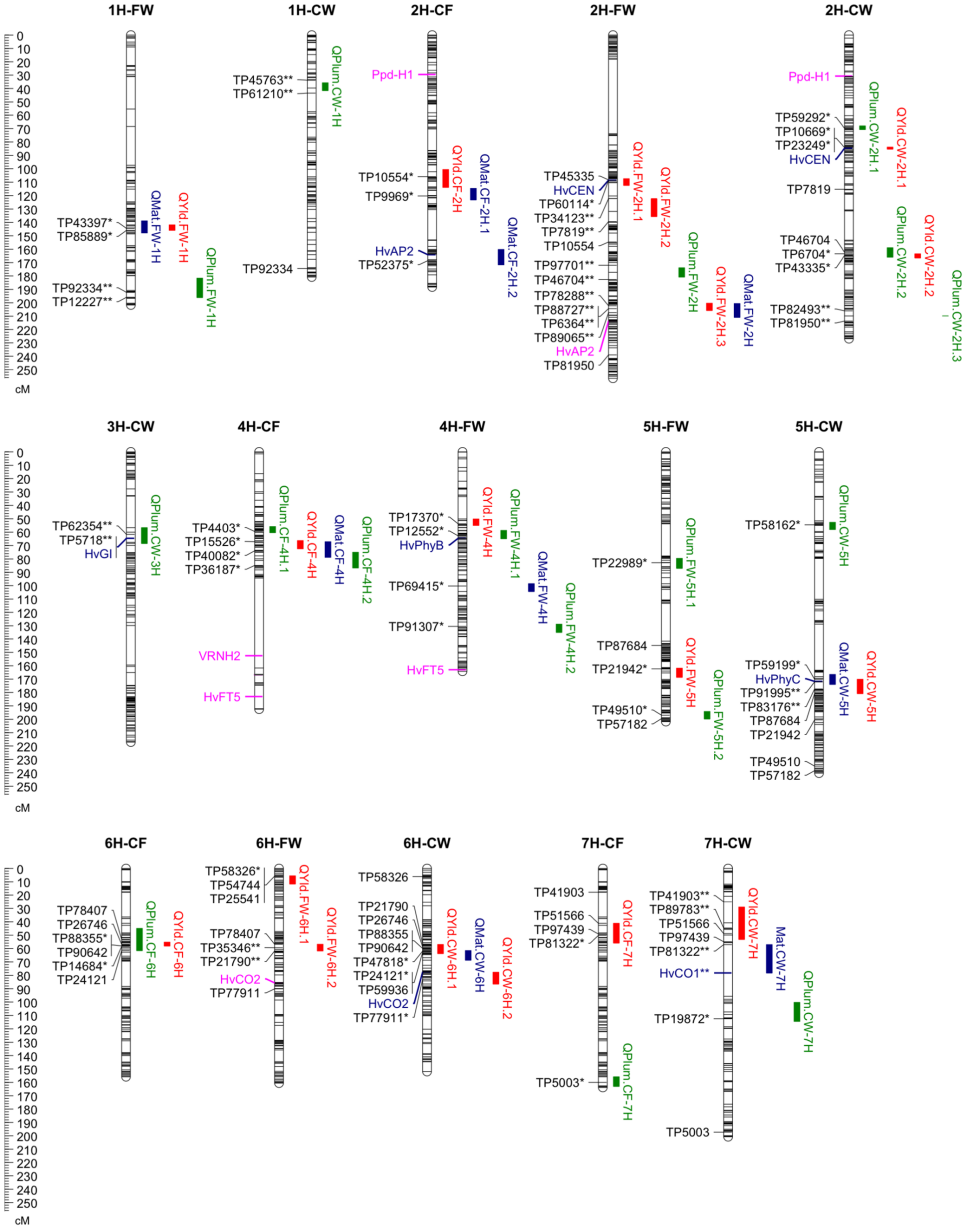
Yield, grain plumpness and maturity QTL positions in the CF, CW and FW populations. * = closest marker to the QTL peak. ** = markers flanking a QTL peak. Known phenology genes outside of QTL intervals are shown with pink colour, while those co-located with QTL are shown in blue.

Genetic predictors were estimated with a step size of 2 cM interval and the minimum distances for cofactor proximity and for declaring independent QTL were set to 30 cM and 20 cM, respectively. Repeated iterations of CIM were performed until no further change in the selected QTL was observed [14]. QTL main effects, QTL x Environment interaction effects, percent of phenotypic variance explained by the QTL (PVE) and the source of high value allele at each environment were determined for all significant QTL remaining in the final QTL model. Results were presented in Fig 1, Tables 2 and 3.

**Table 2:** Yield QTL in three doubled haploid populations of barley at six environments in southern Australia.

QTL	Significant marker	Chr.	Position (cM)	LOD	QTL x E	PVE (%)	QTL additive effects (t/ha)					
							MRC12	MRC13	RAC12	RAC13	SWH12	SWH13
*QYld*.*CF-2H*	TP10554	2H	105.9	4.2	yes	2.6–8.2	-	-	0.11^C^	-	-	0.06^C^
*QYld*.*CF-4H*	TP15526	4H	67.1	4.2	yes	1.8–9.1	-	0.08^C^	-	-	0.05^C^	0.05^C^
*QYld*.CF-6H*	TP88355	6H	58.1	14.7	yes	6.1–25.0	-	0.06^F^	-	0.23^F^	-	-
*QYld*.CF-7H*	TP81322	7H	50.2	4.0	yes	1.5–8.5	-	-	-	0.06^C^	0.07^C^	0.08^C^
*QYld*.*CW-2H*.*1*	TP23249	2H	84.2	15.3	yes	4.6–24.4	0.04^C^	-	0.10^C^	-	0.16^C^	0.08^C^
*QYld*.*CW-2H*.2*	TP43335	2H	164.6	6.9	yes	4.2–9.6	0.06^W^	0.06^W^	-	-	-	-
*QYld*.*CW-5H*	TP91995-TP83176 ^#^	5H	173.9	3.7	yes	2.0–5.3	0.04^C^	-	-	0.07^W^	-	-
*QYld*.*CW-6H*.*1*	TP24121	6H	62.7	2.9	yes	8.8	-	-	-	-	-	0.11^W^
*QYld*.*CW-6H*.*2*	TP77911	6H	83.0	2.9	yes	4.0–6.0	-	0.06^W^	-	0.13^W^	-	-
*QYld*.CW-7H*	TP41903- TP89783 ^#^	7H	40.7	4.2	yes	2.7–6.0	-	0.06^C^	0.06^C^	0.09^C^	0.08^C^	0.07^C^
*QYld*.*FW-1H*	TP43397	1H	144.5	4.8	yes	4.9–5.5	-	0.08^F^	-	-	-	0.07^F^
*QYld*.*FW-2H*.*1*	TP60114	2H	108.6	6.0	no	2.6–9.3	0.06^F^	0.06^F^	0.06^F^	0.06^F^	0.06^F^	0.06^F^
*QYld*.*FW-2H*.2*	TP34123-TP7819 ^#^	2H	131.8	3.8	yes	7.8	-	-	0.11^W^	-	-	-
*QYld*.*FW-2H*.*3*	TP78288-TP88727 ^#^	2H	203.3	7.7	yes	2.2–4.0	-	-	0.06^W^	-	0.07^F^	0.05^W^
*QYld*.*FW-4H*	TP17370	4H	53.7	5.3	yes	1.2–5.1	-	-	-	0.09^F^	-	0.03^F^
*QYld*.FW-5H*	TP21942	5H	162.3	3.5	yes	4.8	0.05^F^	-	-	-	-	-
*QYld*.*FW-6H*.1*	TP58326	6H	5.8	5.3	yes	2.4–7.7	-	0.08^F^	0.06^F^	-	-	0.08^F^
*QYld*.*FW-6H*.2*	TP35346-TP21790 ^#^	6H	60.6	9.1	yes	10.3	-	0.11^F^	-	-	-	-

* QTL that don’t overlap with maturity QTL or phenology genes;

^#^ the QTL peak is between the indicated markers;

Chr. = chromosome; LOD = logarithm of the odds; QTL x E = QTL x environment interaction; PVE = percent of variance explained by the QTL; “-”: no significant QTL detected in that environment; the superscript letters represent the source of the high value allele (C = Commander, F = Fleet, W = WI4304).

**Table 3:** QTL for grain plumpness in three doubled haploid populations of barley at six environments in southern Australia.

QTL	Significant marker	Chr.	Position (cM)	LOD	QTL x E	PVE (%)	QTL additive effects (% >2.5 mm)				
							MRC13	RAC12	RAC13	SWH12	SWH13
*QPlum*.*CF-4H*.1*	TP4403	4H	59.5	8.3	yes	13.8	-	-	1.98^F^	-	-
*QPlum*.*CF-4H*.*2*	TP36187	4H	84.8	4.8	yes	5.7–9.8	-	-	1.27^C^	2.41^C^	-
*QPlum*.CF-6H*	TP14684	6H	58.4	5.5	yes	2.5–10.9	1.14^F^	2.24^F^	1.76^F^	-	0.54^F^
*QPlum*.CF-7H*	TP5003	7H	160.0	4.3	yes	2.0–10.9	1.47^F^	2.55^F^	0.76^F^	2.53^F^	0.63^F^
*QPlum*.CW-1H*	TP45763-TP36876 ^#^	1H	39.6	4.1	yes	3.1–8.4	2.62^C^	-	1.67^C^	2.23^C^	0.60^C^
*QPlum*.*CW-2H*.1*	TP59292	2H	69.8	14.2	yes	3.0–8.2	1.82^W^	2.11^W^	2.05^W^	-	0.60^C^
*QPlum*.*CW-2H*.2*	TP6704	2H	163.4	5.3	yes	3.1–8.2	2.58^C^	2.76^C^	2.06^C^	2.65^C^	0.60^C^
*QPlum*.*CW-2H*.3*	TP82493-TP81950 ^#^	2H	209.8	4.2	yes	7.4	-	-	-	3.02^W^	-
*QPlum*.*CW-3H*	TP62354-TP5718 ^#^	3H	63.1	5.0	yes	1.6–9.8	-	-	0.91^C^	3.48^C^	-
*QPlum*.CW-5H*	TP58162	5H	54.5	2.8	yes	2.2–6.8	2.35^C^	1.72^C^	-	-	-
*QPlum*.CW-7H*	TP19872	7H	112.5	2.5	yes	1.8–3.5	1.20^C^	2.19^C^	1.20^C^	-	-
*QPlum*.FW-1H*	TP92334-TP12227 ^#^	1H	194.0	4.1	yes	1.5–7.5	1.03^F^	1.83^F^	-	0.95^F^	1.60^F^
*QPlum*.FW-2H*	TP97701-TP46704 ^#^	2H	177.3	11.1	yes	7.0–10.8	2.28^F^	-	1.19^F^	2.08^F^	1.81^F^
*QPlum*.*FW-4H*.*1*	TP12552	4H	62.7	3.1	no	0.3–2.8	0.70^F^	0.70^F^	0.70^F^	0.70^F^	0.70^F^
*QPlum*.*FW-4H*.2*	TP91307	4H	130.5	10.8	yes	2.2–13.7	1.28^W^	3.48^W^	0.62^W^	2.58^W^	2.15^W^
*QPlum*.*FW-5H*.1*	TP22989	5H	82.8	4.4	yes	3.5–5.7	1.65^F^	2.35^F^	0.92^F^	-	-
*QPlum*.*FW-5H*.2*	TP49510	5H	196.9	5.9	yes	1.9–7.4	1.69^F^	1.76^F^	0.88^F^	2.15^F^	-

* QTL that don’t overlap with maturity QTL or phenology genes;

^#^ the QTL peak is between the indicated markers;

Chr. = chromosome; LOD = logarithm of the odds; QTL x E = QTL x environment interaction; PVE = percentage of variance explained by the QTL; “-” = no significant QTL detected in that environment; the superscript letters represent the source of the high value allele (C = Commander, F = Fleet, W = WI4304).

An alternative QTL analysis using grain yield means adjusted for maturity was performed to detect yield QTL independent of the maturity effect. Adjustment for maturity was done by covariance analysis using the spatially adjusted BLUEs as a variate and the Zadok’s score as a covariate. Results were presented in Supplemental S7 Table.

## Results

### Variations in grain yield and grain plumpness

Highly significant (P<0.001) yield differences were observed between the parents of the DH lines in five environments (MRC12, MRC13, RAC13, SWH12 and SWH13), while it was not significant in RAC12 (Table 1). Commander and Fleet yielded equally and more than WI4304 except at RAC13 where WI4304 yield was higher (Table 1). The DH lines showed transgressive segregation for yield in all the three populations (Table 1 and S3, S4 and S5 Figs). The heritability for yield ranged from 0.39 to 0.86.

MRC12 was the lowest yielding environment with the mean yield of 1.26 t/ha in the CF population, 1.00 t/ha in the CW population, and 1.07 t/ha in the FW population. MRC13 was the highest yielding environment for the CF and FW populations, the mean yields of both populations being 3.44 t/ha. RAC12 was the highest yielding environment for the CW population with a mean yield of 3.53 t/ha (Table 1). The genetic correlations among environments for grain yield were positive for all populations and range from a weak correlation between RAC13 and SWH13 in the CF population (r = 0.14) to a moderate correlation between RAC12 and SWH12 in the FW population (r = 0.73) (S5 Table).

Highly significant differences (P<0.001) were observed among the parental genotypes for grain plumpness in all environments except in RAC12 (Table 1). Fleet had higher grain plumpness than Commander and WI4304 in all environments. Commander had more plump grains than WI4304 in MRC13 and SWH12, while the inverse was true in RAC13, and they were equivalent in SWA13 (Table 1). The DH lines in each population showed moderate to high heritability and a wide range of variation for grain plumpness (Table 1).

### Yield QTL

Eighteen QTL were detected for yield across the three populations. All QTL except one on chromosome 2H had significant QTL x environment interactions (Table 2). Yield QTL common between two populations were found on chromosomes 2H and 7H (Fig 1), while a yield QTL on 6H (Fig 1) was detected in all the three populations.

Four QTL were detected in the CF population on chromosomes 2H, 4H, 6H and 7H. Commander contributed the higher value allele for all these QTL except *QYld*.*CF-6H* where the allele was contributed by Fleet. *QYld*.*CF-2H* and *QYld*.*CF-6H* were expressed in two sites while *QYld*.*CF-4H* and *QYld*.*CF-7H* were expressed at three environments, all showing QTL x environment interaction. The QTL *QYld*.*CF-6H*, with the significant marker at the peak LOD score being TP88355, explained 25% and 6.1% of the total phenotypic variance for yield in RAC13 and MRC13, respectively (Table 2). In terms of the actual allele effect on phenotypic value, the Fleet allele increased yield by 3.5% and 16.6%, respectively at MRC13 and RAC13.

Six QTL were detected in the CW population on 2H, 5H, 6H and 7H. Commander contributed the high value allele for *QYld*.*CW-2H*.*1* and *QYld*.*CW-7H* while WI4304 was the high value allele for *QYld*.*CW-2H*.*2*, *QYld*.*CW-6H*.*1* and *QYld*.*CW-6H*.*2*. The QTL *QYld*.*CW-5H* was co-located with the phenology gene *HvPhyC* (Fig 1). *QYld*.*CW-2H*.1 had the highest LOD score of 15.3 and was expressed in four environments (MRC12, RAC12, SWH12 and SWH13) explaining from 4.6% to 24.4% of the phenotypic variance for yield. The QTL on 7H, *QYld*.*CW-7H*, was expressed in five of the six environments with 2.7% to 6.0% of explained phenotypic variance (Table 2).

Eight QTL were detected in the FW population on chromosomes 1H, 2H, 4H, 5H and 6H. The high value alleles for five of these QTL (*QYld*.*FW-2H*.*1*, *QYld*.*FW-4H*, *QYld*.*FW-5H*, *QYld*.*FW-6H*.*1* and *QYld*.*FW-6H*.*2*) were from Fleet while WI4304 contributed the higher value allele for *QYld*.*FW-2H*.*2*. Both Fleet and WI4304 contributed the higher value alleles for *QYld*.*FW-1H* and *QYld*.*FW-2H*.*3* at different environments. The QTL *QYld*.*FW-2H*.*1* at 108.6 cM on 2H, with a LOD score of 6.0, was expressed in all the six environments with no QTL x environment interaction and explained between 2.6% to 9.3% of the total phenotypic variation for yield.

### Grain plumpness QTL

Seventeen QTL were detected for grain plumpness across the three populations: four QTL in CF, seven QTL in CW and six QTL in FW (Table 3 and Fig 1). All QTL except one on 4H in the FW showed significant QTL x environment interaction (Table 3).

Two QTL (*QPlum*.*CF-4H*.*1* and *QPlum*.*CF-4H*.*2*) were detected at 59.5 and 84.8 cM on 4H in CF population. *QPlum*.*CF-4H*.*1* was detected only at RAC13 with the higher value allele from Fleet explaining 13.8% of the phenotypic variance for grain plumpness. *QPlum*.*CF-4H*.*2* was detected at RAC13 and SWH12 with the higher value allele from Commander explaining 5.7% and 9.8% of phenotypic variance, respectively. *QPlum*.*CF-6H* at 58.4 cM was detected in all environments except SWH12 with the higher value allele from Fleet and explaining from 2.5% to 10.9% of the phenotypic variance. *QPlum*.*CF-7H* was detected in all environments with the higher value allele from Fleet increasing the percentage of plump grains by 0.76 to 2.55% (Table 3).

Grain plumpness QTL in the CW population were detected on chromosomes 1H, 2H, 3H, 5H and 7H. *QPlum*.*CW-1H* was detected in all environments except in RAC12 with the higher value allele from Commander explaining 3.1% to 8.4% of the phenotypic variance. Three QTL on 2H (*QPlum*.*CW-2H*.*1*, *QPlum*.*CW-2H*.*2* and *QPlum*.*CW-2H*.*3*) were detected at 69.8 cM, 163.4 cM and 209.8 cM, respectively, and explained from 3.0% to 8.2% of the phenotypic variance (Table 3). Commander contributed the higher value alleles for *QPlum*.*CW-2H*.*1* and *QPlum*.*CW-2H*.*3*, while WI4304 contributed higher value allele for *QPlum*.*CW-2H*.*2* (Table 3). *QPlum*.*CW-3H* explained 1.6% and 9.8% of phenotypic variance at RAC13 and SWH12, respectively. *QPlum*.*CW-5H* was detected in MRC13 and RAC12, explaining 2.2% and 6.8% of phenotypic variance, respectively. *QPlum*.*CW-7H* was detected in MRC13, RAC12 and RAC13, explaining 1.8% to 3.5% of the phenotypic variance. The higher value allele for *QPlum*.*CW-3H*, *QPlum*.*CW-5H*, and *QPlum*.*CW-7H* was contributed by Commander (Table 3).

QTL for grain plumpness in the FW population were detected on chromosomes 1H, 2H, 4H, and 5H, explaining from 3.1% (*QPlum*.*FW-4H*.*1*) to 11.1% (*QPlum*.*FW-2H*) of the phenotypic variance. *QPlum*.*FW-1H*, *QPlum*.*FW-2H*, and *QPlum*.*FW-5H*.*2* were detected in four environments while *QPlum*.*FW-5H*.*1* was detected in three environments. *QPlum*.*FW-4H*.*1* and *QPlum*.*FW-4H*.*2* were detected in all five environments. *QPlum*.*FW-4H*.*1* had the same additive effect across the five environments with no QTL x Environment interaction. Fleet contributed the high value allele for all QTL detected in the FW population except *QPlum*.*FW-4H*.*2* (Table 3).

### Maturity effect on yield QTL

To evaluate the maturity effect on yield in these populations, we used the maturity data collected on the same trials [36]. Significant correlations between yield and maturity were observed in some trials (S6 Table). Two methods were used to evaluate the independency of yield QTL toward maturity: (i) by adjusting the yield data for maturity and comparing the QTL results, and (ii) by co-mapping the QTL for maturity and those for yield or grain plumpness.

The first method used a covariance analysis to adjust the yield QTL for maturity effect. The covariance analysis did not significantly change the QTL as shown in S7 Table. Minor changes due to the adjustment QTL included a slight shift in QTL position and the number of environments where a QTL was detected. For example, in the CF population, *QYld*.*CF-4H* was detected in one more environment, while *QYld*.*CF-6H* was detected in two more environments after correction for maturity (S7 Table). In the FW population, one more QTL on 6H (*QYld*.*FW-6H*.*3*) was detected after correction for maturity effect (S7 Table). An important change was the disappearance of the QTL on 5H, *QYld*.*CW-5H* after the adjustment showing its dependency toward maturity. This QTL is also collocated with *HvPhyC* gene which is known to control the phytochrome pathway [34].

The second method consisted in co-mapping the QTL for yield and grain plumpness with the maturity QTL of the same barley populations [36] (Fig 1). Among 18 yield QTL, six loci were collocated with maturity QTL on chromosomes 1H, 2H, 4H, 5H and 6H. Four yield QTL were collocated with phenology genes, *HvCEN* on chromosome 2H, *HvPhyB* on chromosome 4H, *HvPhyC* on chromosome 5H and *HvCO2* on chromosome 6H. Among 17 QTL for grain plumpness, one QTL, *QPlum*.*CF-4H*.*2*, was collocated with a QTL for maturity (Fig 1). Two other QTL were collocated with phenology genes: *QPlum*.*CW-3H* with *HvGI* gene, and *QPlum*.*FW-4H*.*1* with *HvPhyB* gene (Fig 1). In total, 8 yield QTL and 14 QTL for grain plumpness were independent on phenology (those are marked with * in Tables 2 and 3).

## Discussion

### Environment effects on yield and grain plumpness

The parents of the three populations were selected based on their long-term yield performance in southern Australia. Commander and Fleet had stable yields across a range of environments, while WI4304 had low yields in drought-affected environments. In this study, Commander and Fleet had similar yields, significantly higher than WI4304 except for RAC13 where the rankings were reversed (Table 1). The environments showed substantial variation for yield, which was attributed mainly to the rainfall patterns (amount and distribution), and other climatic and edaphic factors [36]. The wide variation observed in yield and grain plumpness in all of the three populations was expected for such quantitative traits due to transgressive segregation. Except one QTL for grain plumpness (*QPlum*.*FW-4H*.*1*) and one QTL for yield (*QYld*.*FW-2H*.*1*), which were consistent across environments, all QTL for the two traits had significant QTL x environment interactions. One QTL on chromosome 2H in CW, and one QTL on chromosome 6H in the CF population had the strongest effects, though their effects were environment specific.

### QTL independent on maturity or phenology genes

Unlike previous yield QTL studies in barley (see Introduction), none of the major developmental genes, including *Ppd-H1*, *Vrn-H1* and *Vrn-H2*, that drive barley adaptation significantly affected grain plumpness and yield in this study. This could be due to the nature of the populations, which were derived from elite x elite crosses to discover QTL that could be deployed in breeding programs targeting Mediterranean type environments. The lack of significant effect on yield QTL after correction for maturity is also consistent with the nature of the populations.

Twenty-two QTL controlling yield or grain plumpness were phenology independent (see * in Tables 2 and 3). Those were not affected by maturity adjustment of yield data, and not collocated with maturity QTL or phenology genes. Some of these QTL may correspond to QTL described in other populations. *QPlum*.*FW-1H*, located towards the telomere of 1HL, was in a similar position in the Galleon x Haruna Nijo barley population [51]. *QPlum*.*CW-1H*, which is on a different region to *QPlum*.*FW-1H*, is around the end of chromosome 1HS where grain plumpness QTL in Blenheim x E224/3, Harrington x Morex, and Chebec x Harrington populations were mapped [6]. The location of the other grain plumpness QTL on chromosome 2H in CW (*QPlum*.*CW-2H*.*3*) seems to coincide with the screenings QTL reported in the Sloop x Alexis population, and thousand grain weight QTL found in the Blenheim x E224/3 population [6]. The grain plumpness QTL detected in CW and FW populations on 5H seems to be at a similar position to the QTL for grain plumpness and screenings in the Chebec x Harrington population [52]. Some QTL were not reported in other populations and are new, such as *QYld*.*CF-2H*, *QYld*.*FW-5H*, *QPlum*.*CW-2H*.*1*.

The two yield QTL detected on chromosome 7H (*QYld*.*CF-7H* and *QYld*.*CW-7H*) have common markers (TP51566 and TP97439) on the genetic map (Fig 1), though they are clearly separated on the barley cv Morex RefSeq v1.0 map (S1 Table). Other studies have reported yield QTL in the same genomic region [23, 53]. Although these two QTL were located around the *Vrn-H3* locus, where [21] have also reported QTL for yield and flowering date, Vrn-H3 marker was monomorphic in our populations and cannot explain the yield QTL.

### Yield and grain plumpness QTL related to maturity

By comparing the QTL for yield and grain plumpness with maturity QTL previously found in these populations [36], we found ten QTL for yield and three QTL for grain plumpness that co-located to maturity QTL and sometimes with known phenology genes suggesting some pleiotropic effects for six regions of the genome:

The yield QTL *QYld*.*FW-1H* on chromosome 1H co-located with the maturity QTL *QMat*.*FW-1H* in the FW population. Two regions on chromosome 2H, *QYld*.*CF-2H and QYld*.*FW-2H*.*3*, also match maturity QTL in CF and FW populations [36]. These regions are 50 Mbp apart on the barley reference genome sequence RefSeq v1.0 (S1 Table).

The peak markers for the yield QTL, *QYld*.*FW-4H* (TP17370), the grain plumpness QTL, *QPlum*.*CF-4H*.*1* (TP4403) and *QPlum*.*FW-4H*.*1* (TP12552) on chromosome 4H are located close to each other on the barley RefSeq v1.0 (S1 Table). These markers are also co-located with TP89118 flanking the maturity QTL *QMat*.*CW-4H* reported in [36]. This suggests this QTL might have pleiotropic effects on yield, grain plumpness and maturity. QTL for plant height, thousand-grain weight, spikes per square metre, and spike morphology were reported around the same genomic region in the Nure x Tremois population [20].

The yield QTL, *QYld*.*CW-5H*, on chromosome 5 disappeared after adjustment for maturity effects (S7 Table). This QTL co-locates with the maturity QTL (*QMat*.*CW-5H*) and leaf waxiness QTL (*QLwax*.*CW-5H*) [36], and aligned on the barley RefSeq v1.0 map with the maturity QTL (*QMat*.*CF-5H*.*2*) in the CF population (S1 Table). In a previous study, different QTL that control reproductive development stages from awn primordia formation to anther extrusion, were mapped to this region [54]. Thus, it appears that this yield QTL is related to a direct effect of maturity. *QYld*.*CW-5H* is closely linked to *HvPhyC* locus (Fig 1), which has a role in promoting long day flowering in barley [55].

On chromosome 6H, Fleet allele increased yield at *QYld*.*CF-6H* in population CF and *QYld*.*FW-6H*.*2* in population FW. Although these QTL don’t overlap with a maturity QTL in these populations, they shared common markers with *QYld*.*CW-6H*.*1* and a maturity QTL (*QMat*.*CW-6H*) in population CW (Fig 1). Adjustment for maturity effects in the QTL analysis increased the PVE from 10.3% to 18.9% for the *QYld*.*FW-6H*.*2* showing a small maturity effect on this yield QTL. Further studies would be necessary to confirm that these QTL are due to the same gene in the three populations.

### QTL co-located with phenology genes without affecting maturity

We identified some QTL for yield or grain plumpness that are co-located with phenology genes but not with a maturity QTL in these populations [36] (S1 Table). Either these phenology genes affect inflorescence development with an impact on yield or grain plumpness without changing the flowering time, or there is an alternate responsible gene near the phenology gene. Such examples were found on chromosomes 2H, 3H and 4H. On chromosome 3H, *QPlum*.*CW-3H* co-located with *HvGI*, the barley homologue of an Arabidopsis photoperiod pathway gene [33]. *QPlum*.*FW-4H*.*1* was collocated with *HvPhyB* gene that control flowering time via the red/far-red light *PHYTOCHROMES* pathway.

We examined the colocation between *HvCEN* gene and the yield QTL *QYld*.*CW-2H*.*1* and *QYld*.*FW-2H*.*1* on chromosome 2H (Fig 1). These two QTL are at the same position on chromosome 2H (Fig 1, S4 Table) and distinct from *QYld*.*CF-2H* (Fig 1). The absence of this QTL in the CF population suggests a common haplotype between Commander and Fleet, with both parents contributing the high yield allele in the CW and FW populations where the QTL was detected. *HvCEN* is the gene for *EPS2* [30], which influences flowering time independently of vernalization and photoperiod [26]. *HvCEN* is associated with a phenology QTL and coincides with yield and grain size QTL [6, 14, 20, 23]. *QYld*.*CW-2H*.*1* and *QYld*.*FW-2H*.*1* are not associated with a difference in maturity in our populations. Sequencing of *HvCEN* in Commander, Fleet and WI4304 revealed seven new sequence variants (S1 Fig, S1 File) that were not previously described in [30]. Fleet and Commander showed different haplotypes for the SNP in promoter region. The only SNP that is common to Fleet and Commander and contrasted with WI4304 is in intron 3 and unlikely to explain the QTL. The *QYld*.*CW-2H*.*1* and *QYld*.*FW-2H*.*1* might be due to another gene closely linked to *HvCEN*.

## Conclusions

Temperature, day length and rainfall are important climatic factors that dictate crop adaptation and distribution. Photoperiod response and vernalization requirement are the major determinants of adaptation in barley and other cereals like wheat, and major genes controlling these traits have been identified. These genes have pleiotropic effects on heading date, plant architecture, yield and other important traits and exert large effects that differentiate the different types of germplasm such as spring versus winter types. These genes tend not to vary within locally adapted elite germplasm; thus, breeding for a particular local environment requires deliberate selection of germplasm that, when crossed, would create new allelic combinations leading to superior high yielding genotypes for the target environment. The work described here was designed with a similar purpose in mind, specifically to identify novel alleles that control yield and adaptive traits of barley in the Mediterranean-type environment of South Australia. The three mapping populations used for this study were developed from inter-crossing well-characterized elite Australian genotypes. Twenty-two QTL for yield and grain plumpness were independent of maturity QTL and phenology genes. The lack of association of the major phenology genes (*Ppd-H1*, *Ppd-H2*, *Vrn-H1*, *Vrn-H2* and *Vrn-H3*/*HvFT1*) with yield and grain plumpness here supports the objective for which the parents were selected. The yield QTL on chromosomes 2H, 6H and 7H are common between these populations. Such QTL segregating in different genetic backgrounds could be valuable for marker-assisted selection. However, these QTL had their largest effects only in specific environments, which could limit their application for breeding widely adapted varieties. Marker-assisted pyramiding of the significant QTL into a common genetic background may be a useful breeding strategy to develop varieties adapted to the Australian environment.

## Supporting information

S1 Fig Structure of the *HvCEN* gene sequence of the Australian parental lines. The grey boxes are the 5’ upstream and 3’ downstream regions. The exons are represented in green and the introns in blue. Polymorphic SNP between Fleet, Commander and WI4304 are shown by red bars. HvCEN_1780 is the SNP converted into KASP marker for genetic mapping of *HvCEN* gene.
(PDF)

S2 Fig Genetic map of chromosome 2H in the CW (A) and FW (B) populations. (PDF)

S3 Fig Frequency distribution of yield at six environments in the CF population. CF = Commander x Fleet, MRC12 = Minnipa 2012, MRC13 = Minnipa 2013, RAC12 = Roseworthy 2012, RAC13 = Roseworthy 2013, SWH12 = Swan Hill 2012, SWH13 = Swan Hill 2013.
(PDF)

S4 Fig Frequency distribution of yield at six environments in the CW population. CF = Commander x Fleet, MRC12 = Minnipa 2012, MRC13 = Minnipa 2013, RAC12 = Roseworthy 2012, RAC13 = Roseworthy 2013, SWH12 = Swan Hill 2012, SWH13 = Swan Hill 2013.
(PDF)

S5 Fig Frequency distribution of yield at six environments in the FW population. CF = Commander x Fleet, MRC12 = Minnipa 2012, MRC13 = Minnipa 2013, RAC12 = Roseworthy 2012, RAC13 = Roseworthy 2013, SWH12 = Swan Hill 2012, SWH13 = Swan Hill 2013.
(PDF)

S1 File Sequences of phenology genes *HvAP2*, *HvCO1*, *HvFT5*, *HvGI*, *HvPhyB*, *HvPhyC*, *HvTFL1*, *HvCEN* showing the SNP between Commander, Fleet and WI4304 lines. (TXT)

S1 Table Position of the GBS markers, phenology genes and QTL found in this study and [36] onto the barley RefSeq v1.0 of the map-based sequence of cultivar Morex. (XLSX)

S2 Table Polymorphic SNP at phenology genes in the three barley mapping populations. (DOCX)

S3 Table Primer details of *HvCEN* gene for sequencing and genotyping (KASP assay). (DOCX)

S4 Table PCR mix and cycling program for amplification of *HvCEN*. The enzymes used were Taq DNA polymerase (Invitrogen, CA) and Phusion High-fidelity DNA polymerase (Thermofisher Scientific, UK).
(DOCX)

S5 Table Genetic correlations among six environments for yield in CF, CW and FW populations. (DOCX)

S6 Table Phenotypic correlations (r) between maturity and grain yield at six environments in CF, CW and FW populations. (DOCX)

S7 Table Yield QTL after adjustment for maturity score in CF, CW and FW populations. (DOCX)
