# Supplementary material for: Quantitative trait loci for yield and grain plumpness relative to maturity in three populations of barley (Hordeum vulgare L.) grown in a low rain-fall environment
Source: PLoS One. 2017 May 23;12(5):e0178111. doi: 10.1371/journal.pone.0178111 (PMC5441627; doi:10.1371/journal.pone.0178111)
Supplement: S1 Fig — The grey boxes are the 5’ upstream and 3’ downstream regions. The exons are represented in green and the introns in blue. Polymorphic SNP between Fleet, Commander and WI4304 are shown by red bars. HvCEN_1780 is the SNP converted into KASP marker for genetic mapping of HvCEN gene. (PDF) [file pone.0178111.s001.pdf]

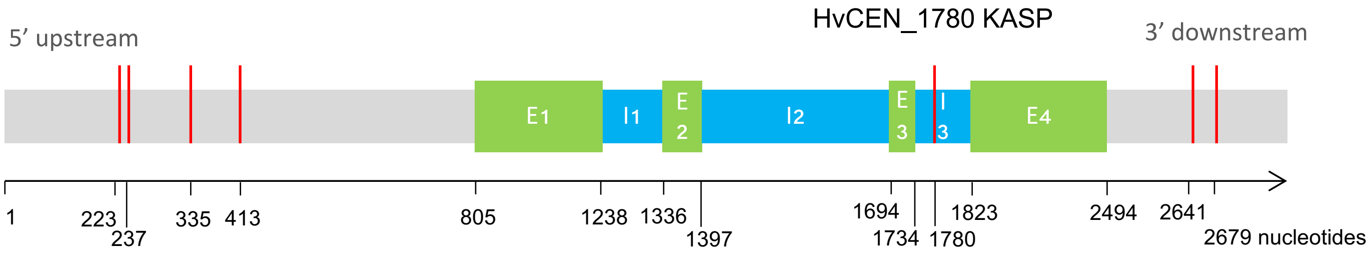

S1 Fig. Structure of the *HvCEN* gene sequence of the Australian parental lines. The grey box are the 5’ upstream and 3’ downstream regions. The exons are represented in green and the introns in blue. Sequence variations between Fleet, Commander and WI4304 are shown by red bars. HvCEN_1780 is the SNP converted into KASP marker for genetic mapping of *HvCEN* gene. Annotation is based on Ensembl MLOC_44160.1.
