# Supplementary figures and images for: Quantitative trait loci for yield and grain plumpness relative to maturity in three populations of barley (Hordeum vulgare L.) grown in a low rain-fall environment
Source: PLoS One. 2017 May 23;12(5):e0178111. doi: 10.1371/journal.pone.0178111 (PMC5441627; doi:10.1371/journal.pone.0178111)

A

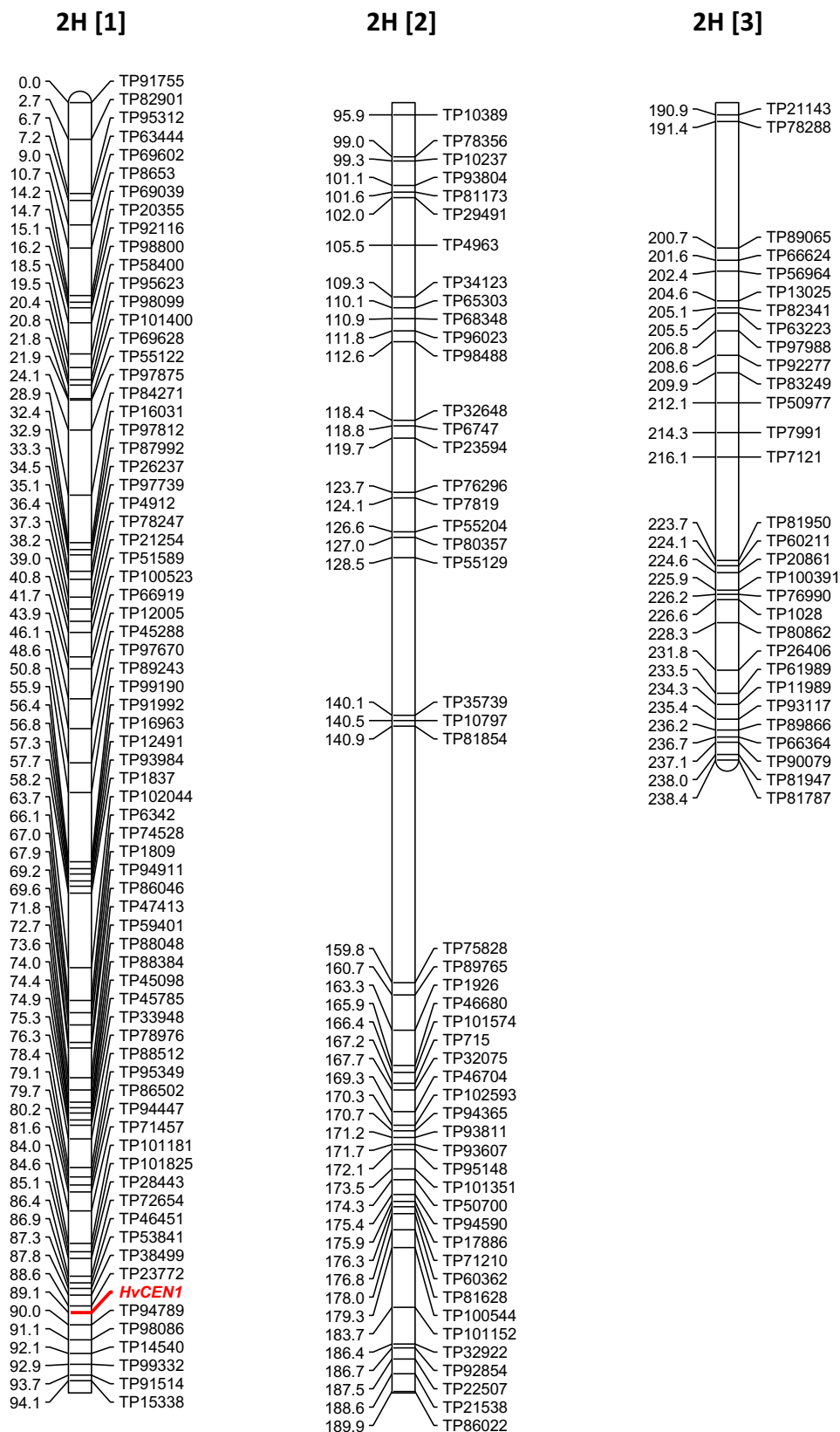

**S2 Fig. Genetic map of chromosome 2H in the CW (A) and FW (B) populations.**

B

2H [1]

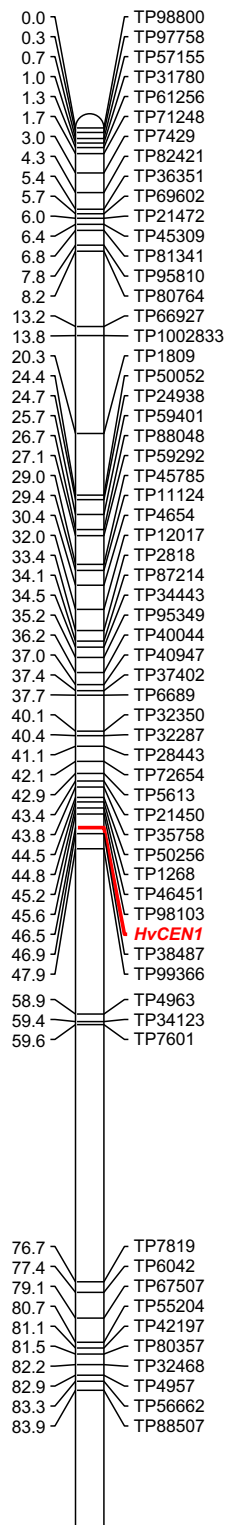

2H [2]

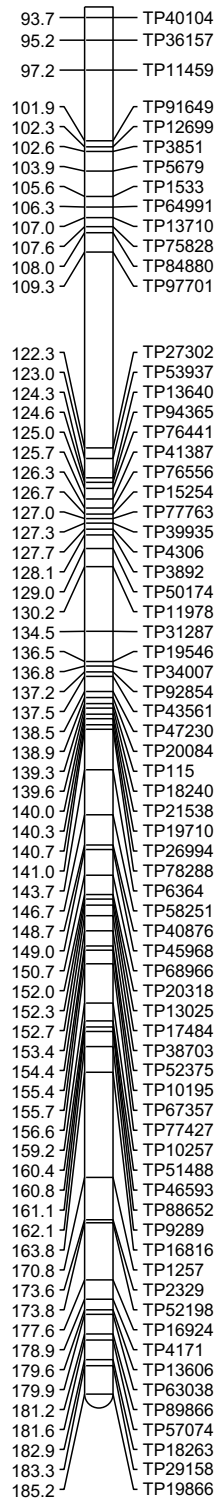

Supplement: S2 Fig — (PDF) [file pone.0178111.s002.pdf]
