# Supplementary material for: Quantitative trait loci for yield and grain plumpness relative to maturity in three populations of barley (Hordeum vulgare L.) grown in a low rain-fall environment
Source: PLoS One. 2017 May 23;12(5):e0178111. doi: 10.1371/journal.pone.0178111 (PMC5441627; doi:10.1371/journal.pone.0178111)
Supplement: S3 Fig — CF = Commander x Fleet, MRC12 = Minnipa 2012, MRC13 = Minnipa 2013, RAC12 = Roseworthy 2012, RAC13 = Roseworthy 2013, SWH12 = Swan Hill 2012, SWH13 = Swan Hill 2013. (PDF) [file pone.0178111.s003.pdf]

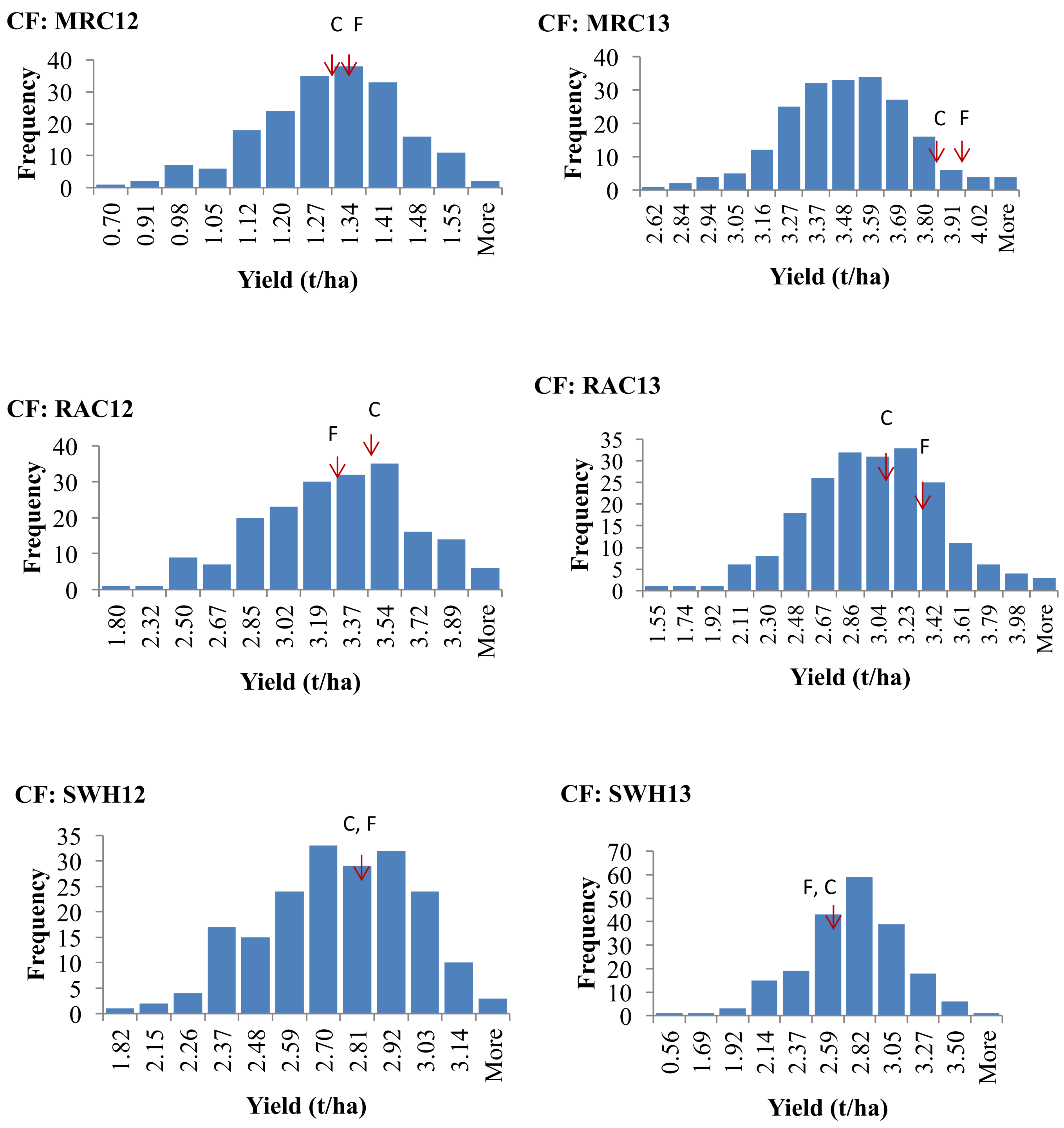

S3 Fig. Histogram of yield at six environments in the CF population. CF= Commander x Fleet, MRC12= Minnipa 2012, MRC13= Minnipa 2013, RAC12= Roseworthy 2012, RAC13= Roseworthy 2013, SWH12= Swan Hill 2012, SWH13= Swan Hill 2013.
