# Supplementary material for: Quantitative trait loci for yield and grain plumpness relative to maturity in three populations of barley (Hordeum vulgare L.) grown in a low rain-fall environment
Source: PLoS One. 2017 May 23;12(5):e0178111. doi: 10.1371/journal.pone.0178111 (PMC5441627; doi:10.1371/journal.pone.0178111)
Supplement: S2 Table — (DOCX) [file pone.0178111.s008.docx]

**S2 Table. Polymorphic SNP at phenology genes in the three barley mapping populations.** P = polymorphic and mapped; M = monomorphic. CF = Commander/Fleet population; CW = Commander/WI4304; FW = Fleet/WI4304.

| SNP ID | Gene | Polymorphism type | Assay | CF | CW | FW |
| --- | --- | --- | --- | --- | --- | --- |
| HvAP2_672 | *HvAP2* | SNP [G/T] | KASP | P | M | P |
| HvCO1_39 | *HvCO1* | SNP [A/G] | KASP | M | P | M |
| HvCO2 | *HvCO2* | SNP [G/A] | HRM | M | P | P |
| HvFT5.1_167 | *HvFT5* | SNP [A/G] | KASP | P | M | P |
| HvGI_3818 | *HvGI* | SNP [C/T] | KASP | M | P | P |
| Ppd-H1 | *Ppd-H1* | SNP [G/A] | HRM | P | P | M |
| HvPhyB_1235 | *HvPhyB* | SNP [A/G] | KASP | M | P | P |
| HvPhyC_3415 | *HvPhyC* | SNP [C/T] | KASP | P | P | M |
| HvTFL1_239 | *HvTFL1* | SNP [C/T] | KASP | P | M | M |
| Vrn-H2 | *Vrn-H2* | Presence/absence | PCR | P | M | M |
| HvCEN_1780 | HvCEN | SNP [C/T] | KASP | M | P | P |
