# Supplementary material for: Quantitative trait loci for yield and grain plumpness relative to maturity in three populations of barley (Hordeum vulgare L.) grown in a low rain-fall environment
Source: PLoS One. 2017 May 23;12(5):e0178111. doi: 10.1371/journal.pone.0178111 (PMC5441627; doi:10.1371/journal.pone.0178111)
Supplement: S3 Table — (DOCX) [file pone.0178111.s009.docx]

**S3 Table. Primer details of *HvCEN* gene for sequencing and genotyping (KASP assay).**

| Primers | Primer sequence | product size (bp) | Aim (polymerase) |
| --- | --- | --- | --- |
| 2541-F | TAGAGGGGCGTTGGTGATAC | 731 | Sequencing (Phusion) |
| 2542-R | CGACATAGGTGGGTTTCAGG |  |  |
| 2533-F | TACCTCAGCTGGGGACTTTG | 787 | Sequencing  (Platinum) |
| 2534-R | GCTCCCTCAGGTATGGATCA |  |  |
| 2535-F | AGTGGTGTCGCTCTTCAGGT | 582 | Sequencing  (Platinum) |
| 2536-R | TCTGTTGGAACAGCACGAAG |  |  |
| 2537-F | GGCCCCATCCTTAATTTCTC | 849 | Sequencing  (Platinum) |
| 2538-R | GGAGGGAGGGAGAACATAGC |  |  |
| 2564-F | TGCAACCTACATCTGCCACT | 544 | Sequencing  (Platinum) |
| 2565-R | CTTAGCCATGGAAAGGGATG |  |  |
| 2566 | GAAGGTGACCAAGTTCATGCTTGCTTTAGCTTTTGCTGATTGGC |  | KASP assay (HvCEN-1780) |
| 2567 | GAAGGTCGGAGTCAACGGATTACTTGCTTTAGCTTTTGCTGATTGGT |  |  |
| 2568 | CTGCATGCATTCATGTGTTTATTCAGAGAA |  |  |
