# Supplementary material for: Quantitative trait loci for yield and grain plumpness relative to maturity in three populations of barley (Hordeum vulgare L.) grown in a low rain-fall environment
Source: PLoS One. 2017 May 23;12(5):e0178111. doi: 10.1371/journal.pone.0178111 (PMC5441627; doi:10.1371/journal.pone.0178111)
Supplement: S4 Table — The enzymes used were Taq DNA polymerase (Invitrogen, CA) and Phusion High-fidelity DNA polymerase (Thermofisher Scientific, UK). (DOCX) [file pone.0178111.s010.docx]

**S4 Table.** **PCR mix and cycling program for amplification of *HvCEN***. The enzymes used were Taq DNA polymerase (Invitrogen, CA) and Phusion High-fidelity DNA polymerase (Thermofisher Scientific, UK).

| **PCR mix using Platinum Taq DNA polymerase** | |
| --- | --- |
| Component | 1 reaction (μl) |
| 10x PCR buffer (-MgCl_2_) | 2.0 |
| 50 mM MgCl_2_ | 0.6 |
| 2.5 mM dNTP | 1.6 |
| Primer F (10 mM) | 0.6 |
| Primer R (10 mM) | 0.6 |
| Platinum Taq polymerase | 0.1 |
| Milli-Q water | 12.5 |
| DNA template (50 ng/μl) | 2.0 |
| **Total** | **20.0μl** |

| **PCR program using Platinum Taq polymerase** | | | |
| --- | --- | --- | --- |
| Step |  | Temperature ( °C) | Time |
| 1 | Denaturation | 94°C | 10 min |
| 2 |  | 94°C | 1 min |
| 3 | Annealing | 70°C | 1 min |
| 4 | Extension | 72°C | 2 min |
| 5 | Step 2 to 4 for 9 cycles decreasing annealing temperature by °C every cycle | | |
| 6 |  | 94°C | 1 min |
| 7 |  | 60°C | 30 sec |
| 8 |  | 72°C | 2 min |
| 9 | Step 6 to 8 for 24 cycles |  |  |
| 10 |  | 72°C | 5 min |

| **PCR mix using Phusion polymerase** | |
| --- | --- |
| Component | 1 reaction (μl) |
| 5x Phusion HF buffer | 4.0 |
| 2.5 mM dNTP | 1.6 |
| Primer F (10 mM) | 1 |
| Primer R (10 mM) | 1 |
| Phusion polymerase | 0.2 |
| Nuclease free water | 10.2 |
| DNA template (50 ng/μl) | 2.0 |
| **Total** | **20.0μl** |

| **PCR program using Phusion polymerase** | | | |
| --- | --- | --- | --- |
| Step |  | Temperature ( °C) | Time |
| 1 | Denaturation | 98°C | 30 sec |
| 2 |  | 98°C | 10 sec |
| 3 | Annealing | 63°C | 20 sec |
| 4 | Extension | 72°C | 25 sec |
| 5 | Step 2 to 4 for 29 cycles | | |
| 6 |  | 72°C | 10 min |
