# Supplementary material for: Quantitative trait loci for yield and grain plumpness relative to maturity in three populations of barley (Hordeum vulgare L.) grown in a low rain-fall environment
Source: PLoS One. 2017 May 23;12(5):e0178111. doi: 10.1371/journal.pone.0178111 (PMC5441627; doi:10.1371/journal.pone.0178111)
Supplement: S5 Table — (DOCX) [file pone.0178111.s011.docx]

**S5 Table. Genetic correlations among six environments for yield in CF, CW and FW populations.**

| Population | Environment | Genetic correlation (r) | | | | | |
| --- | --- | --- | --- | --- | --- | --- | --- |
| CF | MRC12 | 1 |  |  |  |  |  |
|  | MRC13 | 0.45 | 1 |  |  |  |  |
|  | RAC12 | 0.38 | 0.37 | 1 |  |  |  |
|  | RAC13 | 0.21 | 0.21 | 0.18 | 1 |  |  |
|  | SWA12 | 0.52 | 0.51 | 0.43 | 0.24 | 1 |  |
|  | SWA13 | 0.30 | 0.30 | 0.25 | 0.14 | 0.34 | 1 |
|  |  |  |  |  |  |  |  |
| CW | MRC12 | 1 |  |  |  |  |  |
|  | MRC13 | 0.63 | 1 |  |  |  |  |
|  | RAC12 | 0.71 | 0.65 | 1 |  |  |  |
|  | RAC13 | 0.28 | 0.26 | 0.29 | 1 |  |  |
|  | SWH12 | 0.69 | 0.63 | 0.72 | 0.28 | 1 |  |
|  | SWH13 | 0.52 | 0.48 | 0.54 | 0.21 | 0.53 | 1 |
|  |  |  |  |  |  |  |  |
| FW | MRC12 | 1 |  |  |  |  |  |
|  | MRC13 | 0.55 | 1 |  |  |  |  |
|  | RAC12 | 0.60 | 0.62 | 1 |  |  |  |
|  | RAC13 | 0.45 | 0.66 | 0.51 | 1 |  |  |
|  | SWH12 | 0.64 | 0.67 | 0.73 | 0.55 | 1 |  |
|  | SWH13 | 0.51 | 0.67 | 0.58 | 0.61 | 0.62 | 1 |
|  |  |  |  |  |  |  |  |
|  |  | MRC12 | MRC13 | RAC12 | RAC13 | SWH12 | SWH13 |
