# Supplementary material for: Quantitative trait loci for yield and grain plumpness relative to maturity in three populations of barley (Hordeum vulgare L.) grown in a low rain-fall environment
Source: PLoS One. 2017 May 23;12(5):e0178111. doi: 10.1371/journal.pone.0178111 (PMC5441627; doi:10.1371/journal.pone.0178111)
Supplement: S6 Table — (DOCX) [file pone.0178111.s012.docx]

**S6 Table.** **Phenotypic correlations (r) between maturity and grain yield at six environments in CF, CW and FW populations.**

| Population | Environment | | | | | |
| --- | --- | --- | --- | --- | --- | --- |
|  | MRC12 | MRC13 | RAC12 | RAC13 | SWH12 | SWH13 |
| CF | 0.29*** | 0.21*** | 0.15* | 0.18** | 0.13^ns^ | 0.18** |
| CW | 0.42*** | -0.02^ns^ | 0.18** | 0.02^ns^ | -0.04^ns^ | 0.10^ns^ |
| FW | 0.14* | -0.14* | 0.26*** | -0.09^ns^ | 0.2*** | -0.06^ns^ |

*= significant (P< 0.05) **= highly significant (P<0.01), ***= very highly significant (P<0.001), ^ns^ = non-significant
