# Supplementary material for: Quantitative trait loci for yield and grain plumpness relative to maturity in three populations of barley (Hordeum vulgare L.) grown in a low rain-fall environment
Source: PLoS One. 2017 May 23;12(5):e0178111. doi: 10.1371/journal.pone.0178111 (PMC5441627; doi:10.1371/journal.pone.0178111)
Supplement: S7 Table — (DOCX) [file pone.0178111.s013.docx]

**S7 Table. Yield QTL after adjustment for maturity score in CF, CW and FW populations.**

| QTL | Significant marker | Chr. | Position | LOD | QTL x E | PVE (%) | QTL additive effects | | | | | |
| --- | --- | --- | --- | --- | --- | --- | --- | --- | --- | --- | --- | --- |
|  |  |  |  |  |  |  | MRC12 | MRC13 | RAC12 | RAC13 | SWH12 | SWH13 |
| *QYld.CF-2H* | TP10554 | 2H | 105.9 | 4.0 | yes | 2.8-7.4 | - | - | 0.102^C^ | - | - | 0.06^C^ |
| *QYld.CF-4H* | TP40024 | 4H | 64.4 | 3.6 | yes | 1.9-5.6 | - | 0.056^C^ | - | 0.06^C^ | 0.046^C^ | 0.051^C^ |
| *QYld.CF-6H* | TP88355 | 6H | 58.1 | 14.0 | yes | 1.8-21.7 | 0.022^F^ | 0.065^F^ | 0.05^F^ | 0.202^F^ | - | - |
| *QYld.CF-7H* | TP81322 | 7H | 50.2 | 4.4 | yes | 3.9-7.3 | - | - | - | - | 0.062^C^ | 0.07^C^ |
| *QYld.CW-2H.1* | TP5613 | 2H | 82.3 | 15.1 | yes | 4.3-23.0 | 0.04^C^ | - | 0.09^C^ | - | 0.15^C^ | 0.08^C^ |
| *QYld.CW-2H.2* | TP41522 | 2H | 165.0 | 7.5 | yes | 4.4-10.0 | 0.06^W^ | 0.06^W^ | - | - | - | - |
| *QYld.CW-6H.1* | TP24121 | 6H | 62.7 | 3.5 | yes | 3.5-10.2 | 0.03^W^ | - | - | - | - | 0.11^W^ |
| *QYld.CW-6H.2* | TP77911 | 6H | 83.0 | 3.0 | yes | 3.9-6.4 | - | 0.06^W^ | - | 0.13^W^ | - | - |
| *QYld.CW-7H* | TP41903- TP89783 ^#^ | 7H | 40.7 | 4.5 | yes | 2.8-6.5 | - | 0.06^C^ | 0.06^C^ | 0.09^C^ | 0.08^C^ | 0.07^C^ |
| *QYld.FW-1H* | TP92933 | 1H | 146.2 | 6.6 | yes | 1.5-6.5 | 0.027^F^ | 0.08^F^ | - | 0.042^F^ | - | 0.061^F^ |
| *QYld.FW-2H.1* | TP60114 | 2H | 108.6 | 6.0 | no | 2.3-7.4 | 0.05^F^ | 0.05F | 0.05^F^ | 0.05^F^ | 0.05^F^ | 0.05^F^ |
| *QYld.FW-2H.2* | TP34123-TP7819 ^#^ | 2H | 129.8 | 3.3 | yes | 6.8 | - | - | 0.09^W^ | - | - | - |
| *QYld.FW-2H.3* | TP78288-TP88727 ^#^ | 2H | 203.3 | 7.2 | yes | 1.6-5.2 | - | - | 0.044^W^ | - | 0.069^F^ | 0.04^W^ |
| *QYld.FW-4H* | TP17370 | 4H | 53.7 | 5.9 | yes | 2.1-6.3 | - | - | - | 0.09^F^ | - | 0.04^F^ |
| *QYld.FW-5H* | TP100214 | 5H | 162.5 | 4.0 | yes | 1.8-4.4 | 0.04^F^ | - | 0.05^W^ | - | - | - |
| *QYld.FW-6H.1* | TP65356 | 6H | 8.7 | 6.9 | yes | 1.7-9.2 | - | 0.08^F^ | 0.05^F^ | 0.05^F^ | - | 0.08^F^ |
| *QYld.FW-6H.3* | TP77950 | 6H | 37.2 | 3.2 | yes | 2.9-4.0 | 0.03^F^ | 0.06^W^ | - | - | 0.06^F^ | - |
| *QYld.FW-6H.2* | TP35346-TP21790 ^#^ | 6H | 60.6 | 10.2 | yes | 18.9 | - | 0.14^F^ | - | - | - | - |

^#^the actual QTL peak is between the indicated markers. “-” indicates that the QTL no significant QTL was detected in that environment, and the superscript letters represent the source of the high value allele (C= Commander, F= Fleet, W= WI4304). LOD = logarithm of the odds. PVE= percentage of variance explained by the QTL. A range of PVE is given when the QTL is significant in more than one environment.
